# Supplementary material for: Optimizing the Production of gp145, an HIV-1 Envelope Glycoprotein Vaccine Candidate and Its Encapsulation in Guanosine Microparticles
Source: Vaccines (Basel). 2023 May 12;11(5):975. doi: 10.3390/vaccines11050975 (PMC10221277; doi:10.3390/vaccines11050975)
Supplement: Supplementary file 1 [file vaccines-11-00975-s001.zip › vaccines-2322965-supplementary.pdf]

Supplementary Figures

Media base	Estimated titer (μg/mL)	Comments
PowerCHO-1	Not pursued	This culture medium was only used to evaluate CHO-K1 cells and adapt them to PowerCHO-2.
PowerCHO-2	160	This was the culture medium that produced the best titers and was used in our study
PowerCHO-3	Not pursued	Not pursued, unavailable commercial or GMP-grade
PowerCHO Advance	45	Not pursued due to low titers
OptiCHO	158	Not pursued due to high gp145 degradation
FortiCHO	50	Not pursued due to low titers
Dynamis	50	Not pursued due to low titers
BDCHO	75-100	Not pursued, unavailable commercial or GMP-grade

Table S1. Cell culture media tested in the current study. A total of eight different cell culture media bases were tried in our effort to identify the best medium to increase gp145 titers. Our screening indicated that PowerCHO-2 was the medium that produced the highest gp145 titer.

Table S2. pH studies using 0.250L bioreactors

pH setpoint	gp145 titer (mg/L) ± SD
7.2	47 ± 0
7.0	59 ± 2
6.8	86 ± 8
SF	91 ± 11

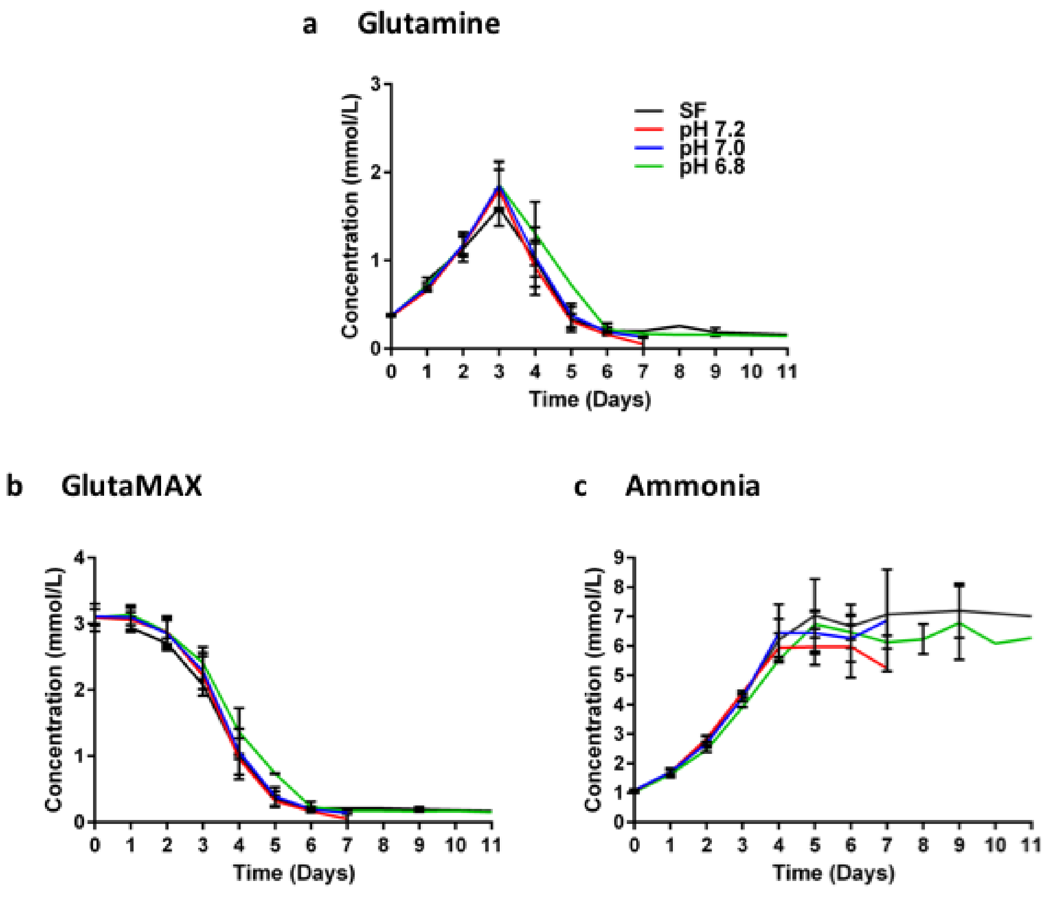

Figure S1. Analysis of metabolites present in cell cultures during pH studiesin 0.250L bioreactors. (a) glutamine levels, (b) GlutaMAX concentration, and (c) ammonia profile. A Cedex Bio Analyzer was employed to measure metabolites from supernatants. In eachfigure the following colors are used: black for control shake flask (SF); red for pH 7.2; blue for pH 7.0; green for pH 6.8.

Table S3. Feed experiments to increase gp145 titer

Condition #	Condition	gp145 Titer (mg/L) ± SD
1	Glucose and GlutaMAX (feed on day 3 and day 6)	123 ± 2
2	Glucose and GlutaMAX feed (glucose between 2–3 g/L)	160 ± 1
3	Glucose feed up to 6 g/L	121 ± 1
N/A	No Feed (control batch)	109 ± 1

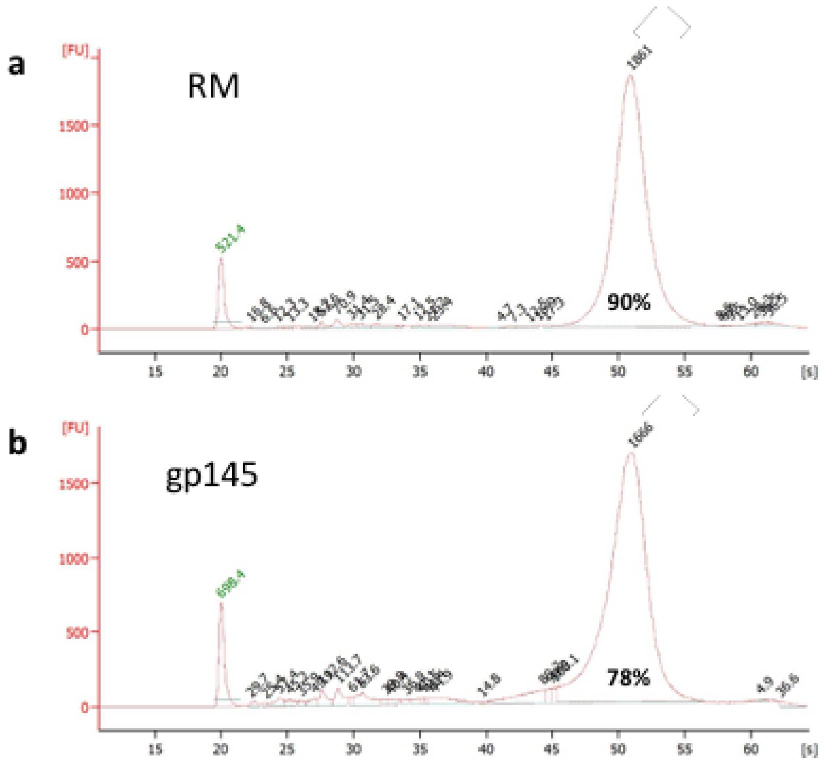

Figure S2. Protein purity analysis by microchip capillary gel electrophoresis.
(a) Electropherogram coming from the MCGE analysis shown in Figure 3c for the reference standard (RM). The largest peak represents the RM, exhibiting a purity of 90%. The other small peaks represent impurities. (b) Electropherogram from the MCGE analysis shown in Figure 3c for gp145. The largest peak represents our gp145, which is 78% pure. Green peaks represent the lower marker peak, which includes a fluorescent dye and excess labeling dye. The Y-axisrepresents fluorescence units (FU) while the X-axis is time in seconds.

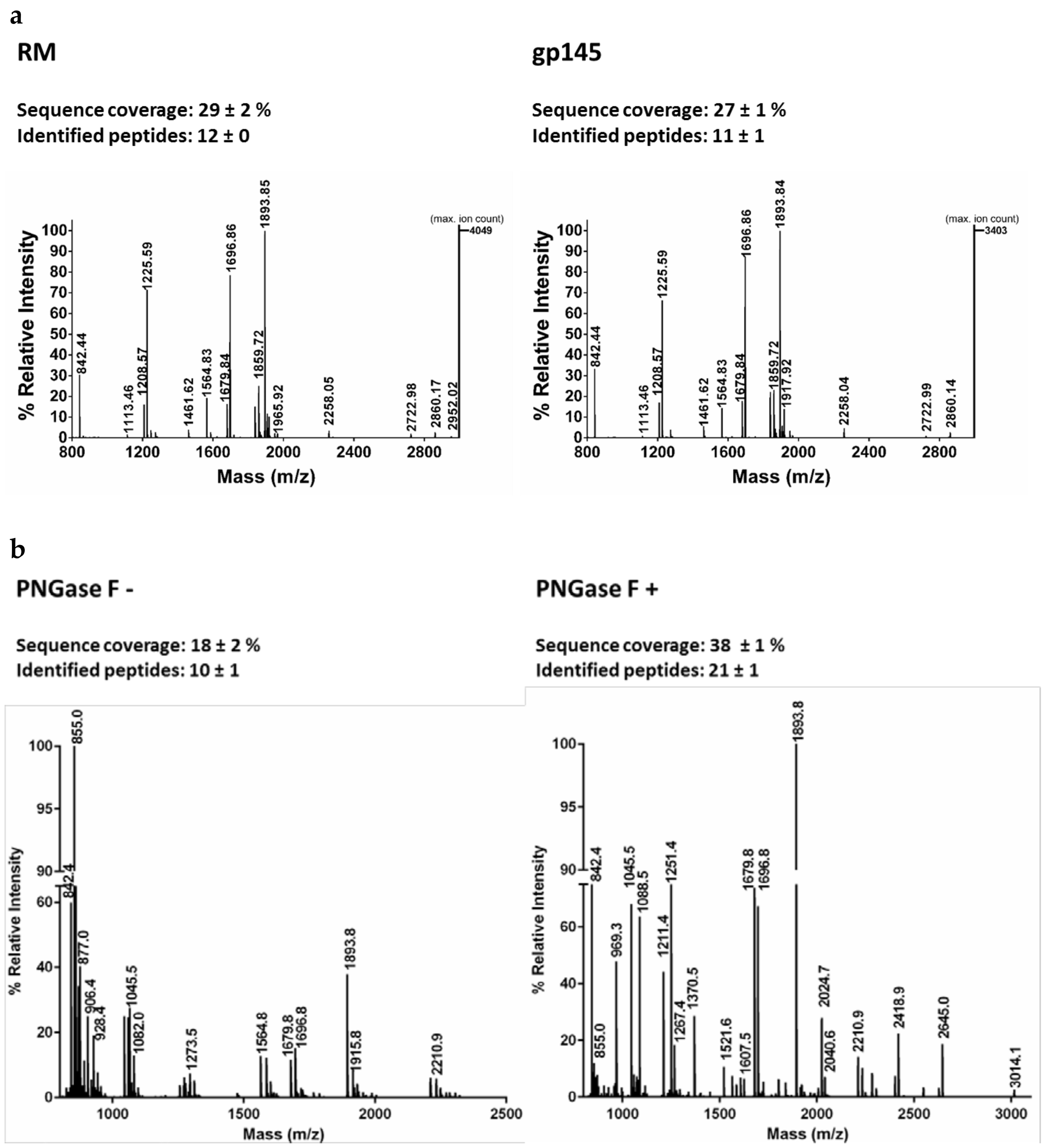

Figure S3. Peptide mass fingerprinting (PMF) of gp145 Env. (a) RM and gp145 have similar PMF profiles. Proteins were not treated with PNGase F. (b) PNGase F treatment (right) increases the PMF coverage relative to untreated (PNGase F-) sample (left). Ten micrograms of RM was used for this analysis. The gp145 was resolved in SDS-PAGE and then excised followed by PNGase F treatment (where indicated), prior to trypsin digestion. Peptides were co-crystallized with α-cyano-4-hydroxycinnamic acid (CHCA) matrix and flown in reflectorpositive mode on the MALDI-TOF.

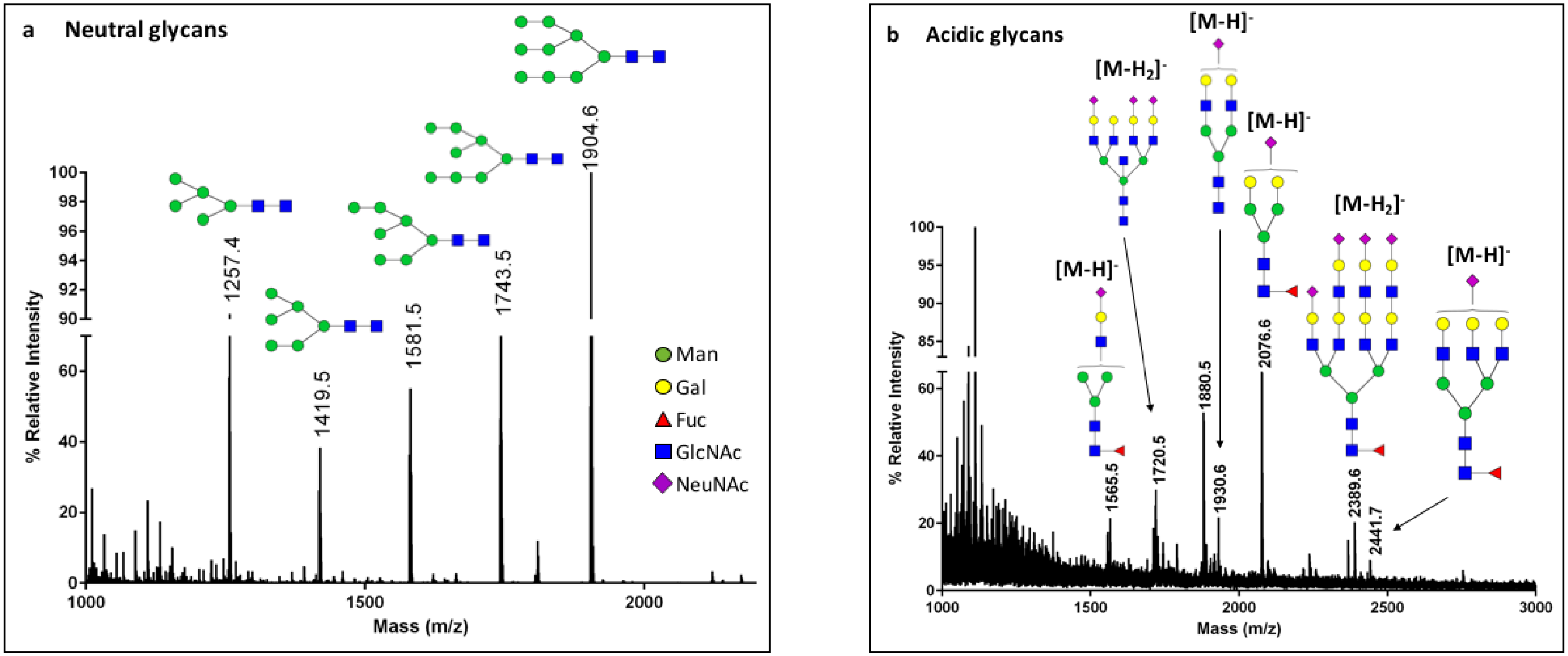

Figure S4. Sample data of N-linked glycans identified by MALDI. (a) Positive Ion MALDI-TOF analysis of neutralN-glycans released from HIV gp145 protein RM. The recombinant gp145 (10 μg) was resolved in an SDS-PAGE and digested with PNGase F. Recovered N-glycans were co-crystalized with THAP matrix. (b) Negative ion MALDI-TOF analysis of acidic N-glycans released from HIV gp145 protein RM. The sample HIV gp145 N-glycans from (a) were used for analysis in the reflector negative mode.Neuraminidase A treatment confirms the glycans which are sialylated [26]. The predicted glycan structures are shown and the mass of the glycans is expressed as m/z in both figures. Mannose (Man), galactose (Gal), fucose (Fuc), N-acetyl glucosamine (GlcNAc), and N-acetyl neuraminic acid or sialic acid (NeuNAc).

Table S4. cIEF percent area analysis

Sample	Group I (%)	Group II (%)	Group III (%)
RM	30 ± 1	32 ± 1	39 ± 1
gp145	31 ± 0	32 ± 0	37 ± 0

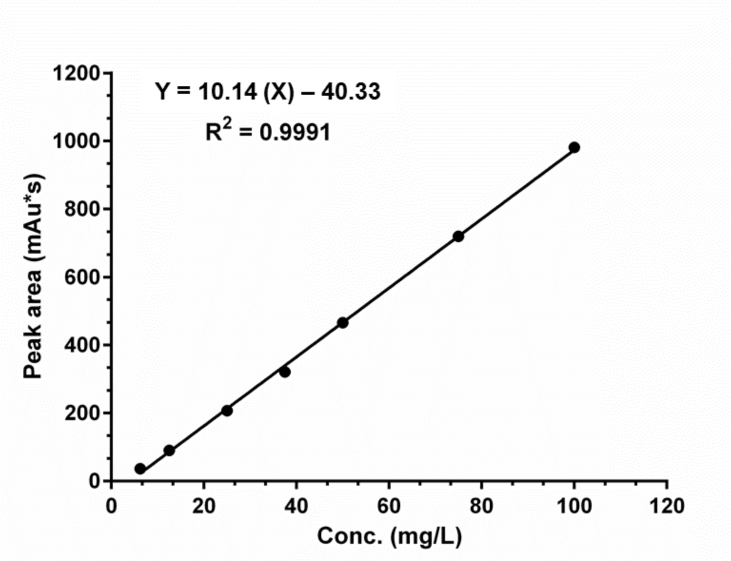

Figure S5. Hydrophobic footprint analysis of gp145 Env. The standard curve employed to calculate the percent recovery. Error on the graph is the standard deviation.

Table S5. DLS size analysis of gp145

Sample	Z-average (d.nm)	PDI	% Intensity
RM	23.5 ± 2.0	0.22 ± 0.06	93 ± 5
gp145	21.5 ± 1.5	0.22 ± 0.05	95 ± 3

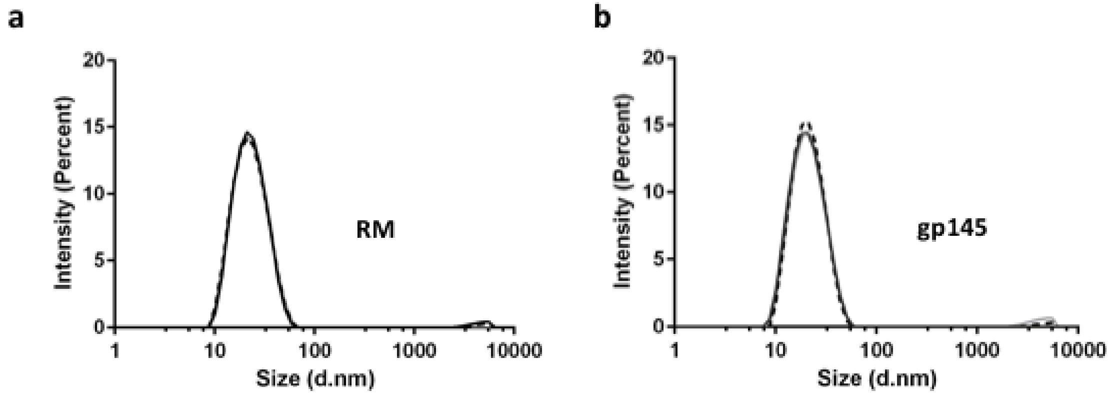

Figure S6. Size analysis of gp145 by dynamic light scattering (DLS). (a) RM gp145 protein analysis via DLS demonstrate size about 23.5 ± 2 d.nm. (b) gp145 analysis throughDLS demonstrate size around 21.5 ± 1.5 d.nm. The three curves in each histogram represent threeindividual measurements. X-axis is in log scale. RM = reference standard

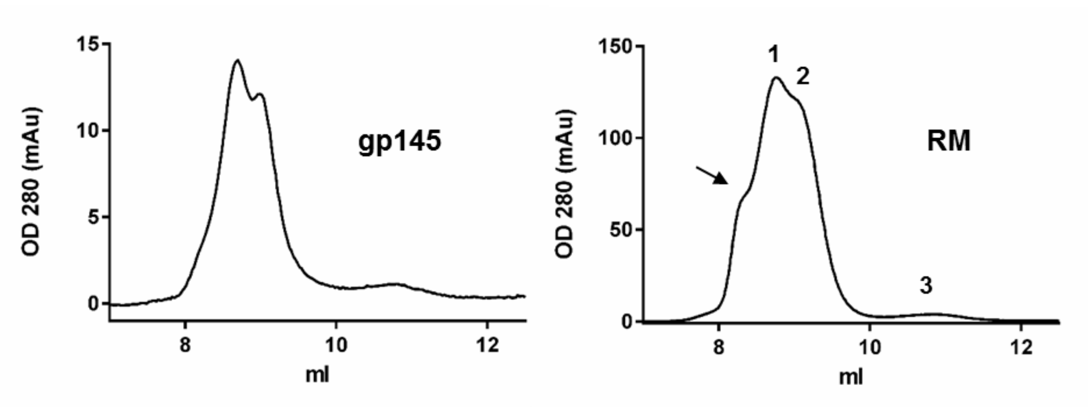

Figure S7. Size Exclusion Chromatography (SEC) Analysis of gp145. The left chromatogram is the profile of our purified gp145; the right chromatogram is that of the reference material (RM). The arrow indicates likely higher order aggregates. The peak numbers indicate fractions that were collected and analyzed by native gel (Figure S8). This analysis was performed using an AKTA FPLC system and a Superdex 200 Increase 10/300 GL column (Cytiva) with 1X PBS as the mobile phase (Sigma tablet, P4417-100TAB). The parameters used for the gp145 run were as follows: 0.15 ml/min flow rate, 50 µl injection volume, 0.34 mg/ml protein concentration. The parameters for the RM run were: 0.7 ml/min flow rate, 100 µl injection volume, and 1mg/ml protein concentration.

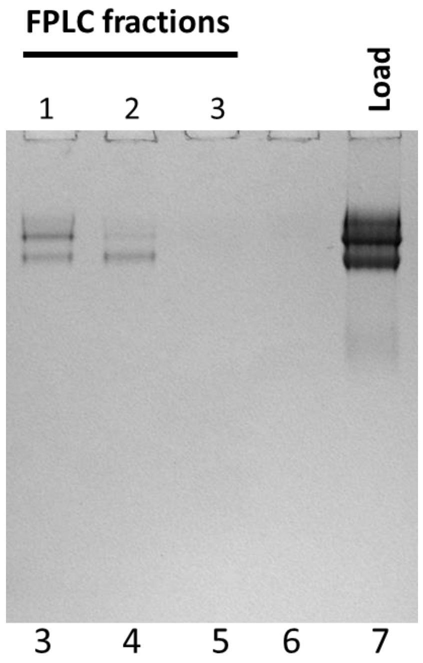

Figure S8. Native gel analysis of RM SEC peaks. 10% TGX precast native PAGE gel (Bio-Rad). The gel is loaded as follows: lane 3, peak 1; lane 4, peak 2; lane 5; peak 3 and lane 7, SEC load.

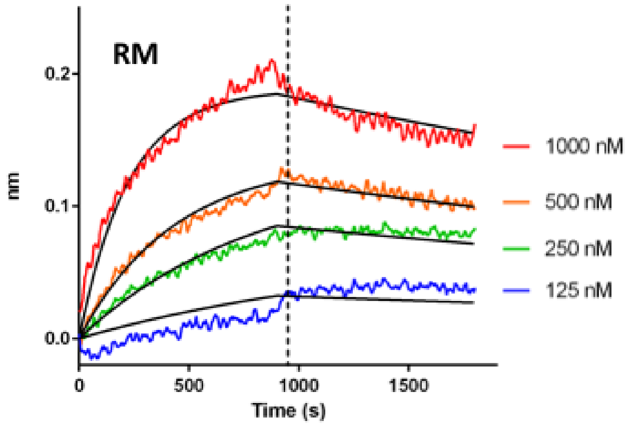

Figure S9. The gp145 RM binding affinity analysis. The RM gp145 binds the broadly neutralizing antibody, 4E10, with a binding affinity of 46 nM. BLI sensorgram generated by binding of serial dilutions of gp145. Black lines represent the 1:1 global fit curves. RM = reference standard.

Table S6. Binding affinity analysis

Sample	K_D (nM)	K_{on} (1/Ms)	K_{dis} (1/S)	Full R^2
RM	46	4.2E+03	2.0E-04	0.979
gp145	65	6.5E+03	4.2E-04	0.979

K_D, dissociation constant; K_{on} , association rate constant; K_{dis} , dissociation rate constant

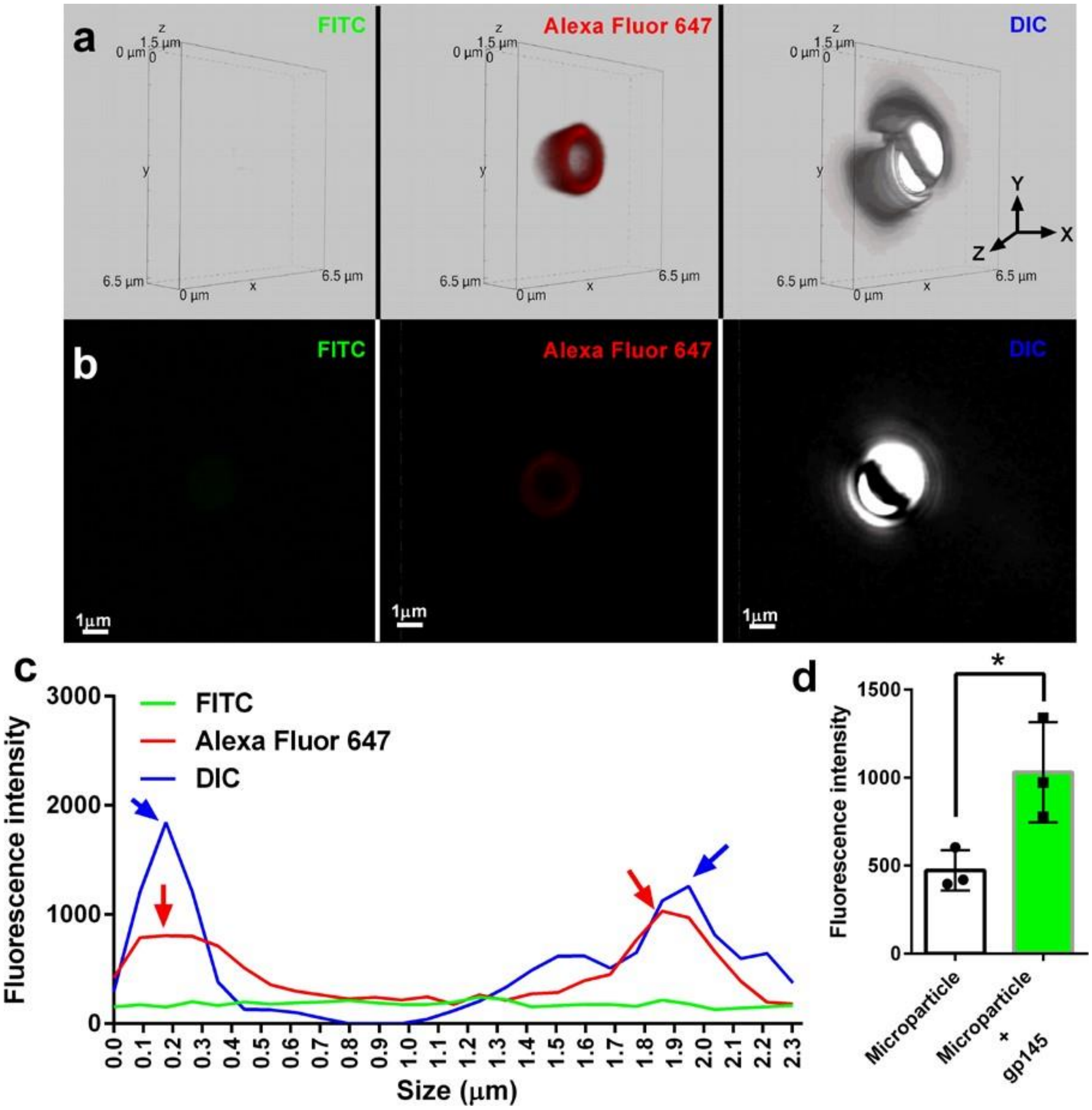

Figure S10. The antibody against VRC01 has negligible binding towards the SHS microparticles. (a) Upper 3D panels, from left to right, show that FITC signal is not detected in microparticles lacking gp145-FITC (far left panel). As can be seen, the secondary antibody exhibits some affinity for the microparticle (middle panel). In the far-right panel, the DIC representation of the microparticles is shown. (b) Two dimensional confocal images demonstrating absence of FITC detection (far left panel), weak signal detection of Alexa Fluor 647 (middle panel), DIC representation (far right panel). (c) Plot showing the DIC borders of the microparticle (blue arrows) and the fluorescence emanating from the secondary antibody recognizing VRC01. Note that the detection of the latter is below the detection of the DIC (red arrows). (d) Although there is some antibody binding to the microparticle, the magnitude of fluorescence after gp145-FITC encapsulation significantly exceeds the (*$p = 0.0348$) non-specific binding of the antibody. There is practically no incorporation of the antibody since its signal (Alexa Fluor 647) practically overlapswith the FITC signal, a fluorophore absent in the experiment (panels a and b on the extreme left). Statistical analysis employed was an unpaired t test.
